# Supplementary material for: An orally administered drug prevents selection for antibiotic-resistant bacteria in the gut during daptomycin therapy
Source: Evol Med Public Health. 2022 Aug 25;10(1):439–46. doi: 10.1093/emph/eoac035 (PMC9472784; doi:10.1093/emph/eoac035)
Supplement: eoac035_Supplementary_Data [file eoac035_supplementary_data.zip › SuppTable1.pdf]

Mouse	Clone	Daptomycin	MIC	Gene	Amino Acid Change	DO (NC_017960.1) Locus Tag	Gene Associated with Daptomycin Susceptibility in E. faecium?
1.1-1	M1.1-1	50 mg/kg	2.5	rapA	Gly891Gly	HMPREF0351_RS11535	no
1.1-1	M1.1-1	50 mg/kg	2.5	hypothetical protein	Ala34Ser	HMPREF0351_RS12370	no
1.2-2	M1.2-2	50 mg/kg	5.5	clsA	Ala20Asp	HMPREF0351_RS05185	yes
1.2-3	M1.2-3	50 mg/kg	6.8	intergenic	NA	upstream HMPREF0351_RS03325	no
1.2-3	M1.2-3	50 mg/kg	6.8	clsA	Ala20Asp	HMPREF0351_RS05185	yes
1.2-4	M1.2-4	50 mg/kg	8	clsA	Ala20Asp	HMPREF0351_RS05185	yes
1.2-5	M1.2-5	50 mg/kg	7.2	clsA	Ala20Asp	HMPREF0351_RS05185	yes
1.4-1	M1.4-1	50 mg/kg	10.7	clsA	Arg218Gln	HMPREF0351_RS05185	yes
1.4-3	M1.4-3	50 mg/kg	9	clsA	Arg218Gln	HMPREF0351_RS05185	yes
1.4-4	M1.4-4	50 mg/kg	11.4	clsA	Arg218Gln	HMPREF0351_RS05185	yes
1.4-5	M1.4-5	50 mg/kg	9.1	clsA	Arg218Gln	HMPREF0351_RS05185	yes
1.8-1	M1.8-1	50 mg/kg	5.2	intergenic	NA	not in DO reference	no
1.8-1	M1.8-1	50 mg/kg	5.2	clsA	Arg211Leu	HMPREF0351_RS05185	yes
1.8-2	M1.8-2	50 mg/kg	4.5	clsA	Arg211Leu	HMPREF0351_RS05185	yes
1.8-3	M1.8-3	50 mg/kg	2.9	intergenic	NA	not in DO reference	no
1.8-3	M1.8-3	50 mg/kg	2.9	infC	Ala143Asp	HMPREF0351_RS01110	no
1.8-3	M1.8-3	50 mg/kg	2.9	yitU	Ile143Val	HMPREF0351_RS07180	no
1.8-5	M1.8-5	50 mg/kg	5.7	clsA	Arg211Leu	HMPREF0351_RS05185	yes
1.10-1	M1.10-1	50 mg/kg	6.8	clsA	Ala20Pro	HMPREF0351_RS05185	yes
1.10-3	M1.10-3	50 mg/kg	5.8	clsA	Arg211Leu	HMPREF0351_RS05185	yes
1.10-4	M1.10-4	50 mg/kg	5.5	clsA	Arg211Leu	HMPREF0351_RS05185	yes
1.10-5	M1.10-5	50 mg/kg	5.5	clsA	Lys61Glu	HMPREF0351_RS05185	yes
1.10-5	M1.10-5	50 mg/kg	5.5	hypothetical protein	Lys109fs	not in DO reference	no
1.12-1	M1.12-1	50 mg/kg	8.4	clsA	Ala20Asp	HMPREF0351_RS05185	yes
1.12-1	M1.12-1	50 mg/kg	8.4	rpsA	Thr404fs	HMPREF0351_RS05365	no
1.12-1	M1.12-1	50 mg/kg	8.4	hypothetical protein	Ser25fs	HMPREF0351_RS12085	no
1.12-2	M1.12-2	50 mg/kg	8.1	hypothetical protein	Arg194Arg	HMPREF0351_RS11625	no
1.12-2	M1.12-2	50 mg/kg	8.1	clsA	Ala20Asp	HMPREF0351_RS05185	yes
1.12-4	M1.12-4	50 mg/kg	6.3	purM	Gly112Gly	HMPREF0351_RS05855	no
1.12-4	M1.12-4	50 mg/kg	6.3	clsA	Ala20Asp	HMPREF0351_RS05185	yes
1.12-5	M1.12-5	50 mg/kg	4.9	hypothetical protein	Arg194Arg	HMPREF0351_RS11625	no
1.12-5	M1.12-5	50 mg/kg	4.9	clsA	Ala20Asp	HMPREF0351_RS05185	yes
1.20-1	M1.20-1	50 mg/kg	6.6	clsA	Arg211Leu	HMPREF0351_RS05185	yes
1.20-3	M1.20-3	50 mg/kg	4.6	clsA	Ala20Asp	HMPREF0351_RS05185	yes
1.20-4	M1.20-4	50 mg/kg	5.9	clsA	Arg211Leu	HMPREF0351_RS05185	yes
1.20-4	M1.20-4	50 mg/kg	5.9	hypothetical protein	Ala519Thr	HMPREF0351_RS09500	no
1.20-5	M1.20-5	50 mg/kg	3.1	clsA	Arg211Leu	HMPREF0351_RS05185	yes
1.24-1	M1.24-1	50 mg/kg	9.4	clsA	Ala20Asp	HMPREF0351_RS05185	yes
1.24-1	M1.24-1	50 mg/kg	9.4	hypothetical protein	Pro24Thr	not in DO reference	no

Mouse	Clone	Daptomycin	MIC	Gene	Amino Acid Change	DO (NC_017960.1) Locus Tag	Gene Associated with Daptomycin Susceptibility in E. faecium?
1.24-1	M1.24-1	50 mg/kg	9.4	HD-domain containing protein	Ser42Tyr	HMPREF0351_RS09100	yes
1.24-2	M1.24-2	50 mg/kg	5.9	clsA	Asn13Ser	HMPREF0351_RS05185	yes
1.24-4	M1.24-4	50 mg/kg	15.6	clsA	Ala20Asp	HMPREF0351_RS05185	yes
1.24-4	M1.24-4	50 mg/kg	15.6	HD-domain containing protein	Leu105Trp	HMPREF0351_RS09100	yes
1.24-5	M1.24-5	50 mg/kg	3.8	clsA	Ala20Asp	HMPREF0351_RS05185	yes
1.24-5	M1.24-5	50 mg/kg	3.8	hypothetical protein	Pro24Thr	not in DO reference	no
1.34-1	M1.34-1	50 mg/kg	2	purD	Ile90Ile	HMPREF0351_RS05870	no
1.34-1	M1.34-1	50 mg/kg	2	ftsH	Ser135Tyr	HMPREF0351_RS12230	no
1.34-5	M1.34-5	50 mg/kg	1.3	whiA	Thr227Ile	HMPREF0351_RS07875	no
1.42-2	M1.42-2	50 mg/kg	3.9	bceB	Ser23Ile	HMPREF0351_RS03555	no
1.42-3	M1.42-3	50 mg/kg	3	bceB	Ser23Ile	HMPREF0351_RS03555	no
1.42-4	M1.42-4	50 mg/kg	2.9	bceB	Ser23Ile	HMPREF0351_RS03555	no
1.42-5	M1.42-5	50 mg/kg	2.8	bceB	Ser23Ile	HMPREF0351_RS03555	no
1.44-1	M1.44-1	50 mg/kg	2.7	pspC	Lys91fs	HMPREF0351_RS09575	yes
1.44-1	M1.44-1	50 mg/kg	2.7	sapB	Ala48Glu	HMPREF0351_RS11315	no
1.44-3	M1.44-3	50 mg/kg	2.5	pspC	Lys91fs	HMPREF0351_RS09575	yes
1.44-3	M1.44-3	50 mg/kg	2.5	sapB	Ala48Glu	HMPREF0351_RS11315	no
1.44-4	M1.44-4	50 mg/kg	2.6	pspC	Lys91fs	HMPREF0351_RS09575	yes
1.44-4	M1.44-4	50 mg/kg	2.6	sapB	Ala48Glu	HMPREF0351_RS11315	no
1.44-5	M1.44-5	50 mg/kg	2.6	pspC	Lys91fs	HMPREF0351_RS09575	yes
1.44-5	M1.44-5	50 mg/kg	2.6	sapB	Ala48Glu	HMPREF0351_RS11315	no
3.2-1	M3.2-1	100 mg/kg	3.4	hypothetical protein	Ile301Thr	HMPREF0351_RS04455	no
3.2-2	M3.2-2	100 mg/kg	3	hypothetical protein	Ile301Thr	HMPREF0351_RS04455	no
3.2-3	M3.2-3	100 mg/kg	6.4	hypothetical protein	Asn146Ser	HMPREF0351_RS01730	no
3.2-3	M3.2-3	100 mg/kg	6.4	hypothetical protein	Ser342Asn	not in DO reference	no
3.2-3	M3.2-3	100 mg/kg	6.4	liaS	Ile301Thr	HMPREF0351_RS04455	yes
3.2-5	M3.2-5	100 mg/kg	2.9	graS	Ala136Asp	HMPREF0351_RS02545	no
3.5-1	M3.5-1	100 mg/kg	1.5	intergenic	NA	not in DO reference	no
3.5-3	M3.5-3	100 mg/kg	7	clsA	Arg211Leu	HMPREF0351_RS05185	yes
3.9-1	M3.9-1	100 mg/kg	3.6	clsA	Ala20Pro	HMPREF0351_RS05185	yes
3.9-1	M3.9-1	100 mg/kg	3.6	hypothetical protein	Leu129fs	not in DO reference	no
3.9-2	M3.9-2	100 mg/kg	5.1	clsA	Ala20Pro	HMPREF0351_RS05185	yes
3.9-2	M3.9-2	100 mg/kg	5.1	hypothetical protein	Leu129fs	not in DO reference	no
3.9-3	M3.9-3	100 mg/kg	5.3	dusB	Ala186Glu	HMPREF0351_RS12220	no
3.9-3	M3.9-3	100 mg/kg	5.3	clsA	Arg211Leu	HMPREF0351_RS05185	yes
3.9-4	M3.9-4	100 mg/kg	6.1	clsA	Ala20Pro	HMPREF0351_RS05185	yes
3.9-4	M3.9-4	100 mg/kg	6.1	hypothetical protein	Leu129fs	not in DO reference	no
3.15-1	M3.15-1	100 mg/kg	5.7	clsA	Ala20Asp	HMPREF0351_RS05185	yes
3.15-1	M3.15-1	100 mg/kg	5.7	hypothetical protein	Val718Val	HMPREF0351_RS08710	no

Mouse	Clone	Daptomycin	MIC	Gene	Amino Acid Change	DO (NC_017960.1) Locus Tag	Gene Associated with Daptomycin Susceptibility in E. faecium?
3.15-2	M3.15-2	100 mg/kg	4.7	clsA	Ala20Asp	HMPREF0351_RS05185	yes
3.15-2	M3.15-2	100 mg/kg	4.7	hypothetical protein	Val718Val	HMPREF0351_RS08710	no
3.15-3	M3.15-3	100 mg/kg	4	clsA	Ala20Asp	HMPREF0351_RS05185	yes
3.15-3	M3.15-3	100 mg/kg	4	hypothetical protein	Val718Val	HMPREF0351_RS08710	no
3.15-4	M3.15-4	100 mg/kg	6.1	intergenic	NA	upstream HMPREF0351_RS03325	no
3.15-4	M3.15-4	100 mg/kg	6.1	clsA	Arg211Leu	HMPREF0351_RS05185	yes
3.15-4	M3.15-4	100 mg/kg	6.1	clsA	Arg211Leu	HMPREF0351_RS05185	yes
3.20-1	M3.20-1	100 mg/kg	3.7	hypothetical protein	Glu284Asp	HMPREF0351_RS07550	no
3.20-1	M3.20-1	100 mg/kg	3.7	dxs	Ile379Ile	HMPREF0351_RS07065	no
3.20-3	M3.20-3	100 mg/kg	3.5	hypothetical protein	Glu284Asp	HMPREF0351_RS07550	no
3.20-3	M3.20-3	100 mg/kg	3.5	dxs	Ile379Ile	HMPREF0351_RS07065	no
3.20-3	M3.20-3	100 mg/kg	3.5	qorB	Glu216Val	HMPREF0351_RS12625	no
3.20-4	M3.20-4	100 mg/kg	2.8	hypothetical protein	Glu284Asp	HMPREF0351_RS07550	no
3.20-5	M3.20-5	100 mg/kg	4	clsA	Ala20Asp	HMPREF0351_RS05185	yes
3.24-2	M3.24-2	100 mg/kg	5.4	clsA	Ala20Asp	HMPREF0351_RS05185	yes
3.24-2	M3.24-2	100 mg/kg	5.4	hypothetical protein	Leu194Ile	HMPREF0351_RS09845	no
3.24-3	M3.24-3	100 mg/kg	5.8	clsA	Ala20Asp	HMPREF0351_RS05185	yes
3.24-3	M3.24-3	100 mg/kg	5.8	hypothetical protein	Leu194Ile	HMPREF0351_RS09845	no
3.24-4	M3.24-4	100 mg/kg	5.9	clsA	Ala20Asp	HMPREF0351_RS05185	yes
3.24-4	M3.24-4	100 mg/kg	5.9	hypothetical protein	Leu194Ile	HMPREF0351_RS09845	no
3.24-5	M3.24-5	100 mg/kg	7.3	hypothetical protein	Leu194Ile	HMPREF0351_RS09845	no
3.24-5	M3.24-5	100 mg/kg	7.3	clsA	Ala20Asp	HMPREF0351_RS05185	yes
3.25-1	M3.25-1	100 mg/kg	4	folD	Gly252Gly	HMPREF0351_RS03705	no
3.25-1	M3.25-1	100 mg/kg	4	liaX	Ala381Thr	HMPREF0351_RS09580	yes
3.25-2	M3.25-2	100 mg/kg	2.7	folD	Gly252Gly	HMPREF0351_RS03705	no
3.25-2	M3.25-2	100 mg/kg	2.7	liaX	Ala381Thr	HMPREF0351_RS09580	yes
3.25-3	M3.25-3	100 mg/kg	4.6	folD	Gly252Gly	HMPREF0351_RS03705	no
3.25-3	M3.25-3	100 mg/kg	4.6	liaX	Ala381Thr	HMPREF0351_RS09580	yes
3.25-5	M3.25-5	100 mg/kg	3.1	folD	Gly252Gly	HMPREF0351_RS03705	no
3.25-5	M3.25-5	100 mg/kg	3.1	liaX	Ala381Thr	HMPREF0351_RS09580	yes
3.28-2	M3.28-2	100 mg/kg	5.4	def	Arg107Ser	HMPREF0351_RS12350	no
3.28-2	M3.28-2	100 mg/kg	5.4	clsA	Arg211Leu	HMPREF0351_RS05185	yes
3.28-2	M3.28-2	100 mg/kg	5.4	hypothetical protein	Trp23Cys	HMPREF0351_RS07365	no
3.28-3	M3.28-3	100 mg/kg	5.3	clsA	Arg211Leu	HMPREF0351_RS05185	yes
3.28-3	M3.28-3	100 mg/kg	5.3	hypothetical protein	Trp23Cys	HMPREF0351_RS07365	no
3.28-4	M3.28-4	100 mg/kg	6.1	clsA	Arg211Leu	HMPREF0351_RS05185	yes
3.28-4	M3.28-4	100 mg/kg	6.1	hypothetical protein	Trp23Cys	HMPREF0351_RS07365	no
3.28-5	M3.28-5	100 mg/kg	5	clsA	Arg211Leu	HMPREF0351_RS05185	yes
3.28-5	M3.28-5	100 mg/kg	5	hypothetical protein	Trp23Cys	HMPREF0351_RS07365	no

Mouse	Clone	Daptomycin	MIC	Gene	Amino Acid Change	DO (NC_017960.1) Locus Tag	Gene Associated with Daptomycin Susceptibility in E. faecium?
3.29-1	M3.29-1	100 mg/kg	10.2	clsA	Arg211Leu	HMPREF0351_RS05185	yes
3.29-1	M3.29-1	100 mg/kg	10.2	intergenic	NA	upstream HMPREF0351_RS09100	no
3.29-3	M3.29-3	100 mg/kg	8.1	clsA	Arg211Leu	HMPREF0351_RS05185	yes
3.29-3	M3.29-3	100 mg/kg	8.1	intergenic	NA	downstream HMPREF0351_RS08120	no
3.29-3	M3.29-3	100 mg/kg	8.1	intergenic	NA	upstream HMPREF0351_RS09100	no
3.29-4	M3.29-4	100 mg/kg	11.5	clsA	Arg211Leu	HMPREF0351_RS05185	yes
3.29-4	M3.29-4	100 mg/kg	11.5	intergenic	NA	downstream HMPREF0351_RS08120	no
3.29-4	M3.29-4	100 mg/kg	11.5	intergenic	NA	upstream HMPREF0351_RS09100	no
3.29-5	M3.29-5	100 mg/kg	3.5	clsA	Arg211Leu	HMPREF0351_RS05185	yes
3.29-5	M3.29-5	100 mg/kg	3.5	intergenic	NA	upstream HMPREF0351_RS09100	no
3.34-1	M3.34-1	100 mg/kg	6.6	dnaB	Gly242Gly	HMPREF0351_RS07485	no
3.34-1	M3.34-1	100 mg/kg	6.6	clsA	Ala20Asp	HMPREF0351_RS05185	yes
3.34-1	M3.34-1	100 mg/kg	6.6	rhaM	Val38Ile	HMPREF0351_RS01040	no
3.34-2	M3.34-2	100 mg/kg	5.7	intergenic	NA	not in DO reference	no
3.34-2	M3.34-2	100 mg/kg	5.7	dnaB	Gly242Gly	HMPREF0351_RS07485	no
3.34-2	M3.34-2	100 mg/kg	5.7	clsA	Ala20Asp	HMPREF0351_RS05185	yes
3.34-4	M3.34-4	100 mg/kg	4.3	dnaB	Gly242Gly	HMPREF0351_RS07485	no
3.34-4	M3.34-4	100 mg/kg	4.3	clsA	Ala20Asp	HMPREF0351_RS05185	yes
3.34-5	M3.34-5	100 mg/kg	6.2	dnaB	Gly242Gly	HMPREF0351_RS07485	no
3.34-5	M3.34-5	100 mg/kg	6.2	clsA	Ala20Asp	HMPREF0351_RS05185	yes
3.34-5	M3.34-5	100 mg/kg	6.2	hypothetical protein	Gly92Glu	HMPREF0351_RS07320	no
3.45-2	M3.45-2	100 mg/kg	4	clsA	Asp100Val	HMPREF0351_RS05185	yes
3.45-2	M3.45-2	100 mg/kg	4	pnp	Gln22Lys	HMPREF0351_RS00785	no
3.45-4	M3.45-4	100 mg/kg	1.7	hypothetical protein	Leu77His	HMPREF0351_RS07080	no
3.45-5	M3.45-5	100 mg/kg	1.5	intergenic	NA	not in DO reference	no
3.45-5	M3.45-5	100 mg/kg	1.5	fabG	Val181Leu	HMPREF0351_RS07375	no
