# Supplementary material for: An orally administered drug prevents selection for antibiotic-resistant bacteria in the gut during daptomycin therapy
Source: Evol Med Public Health. 2022 Aug 25;10(1):439–46. doi: 10.1093/emph/eoac035 (PMC9472784; doi:10.1093/emph/eoac035)
Supplement: eoac035_Supplementary_Data [file eoac035_supplementary_data.zip › Supplementary Figures.docx]

**Supplementary Figures:**


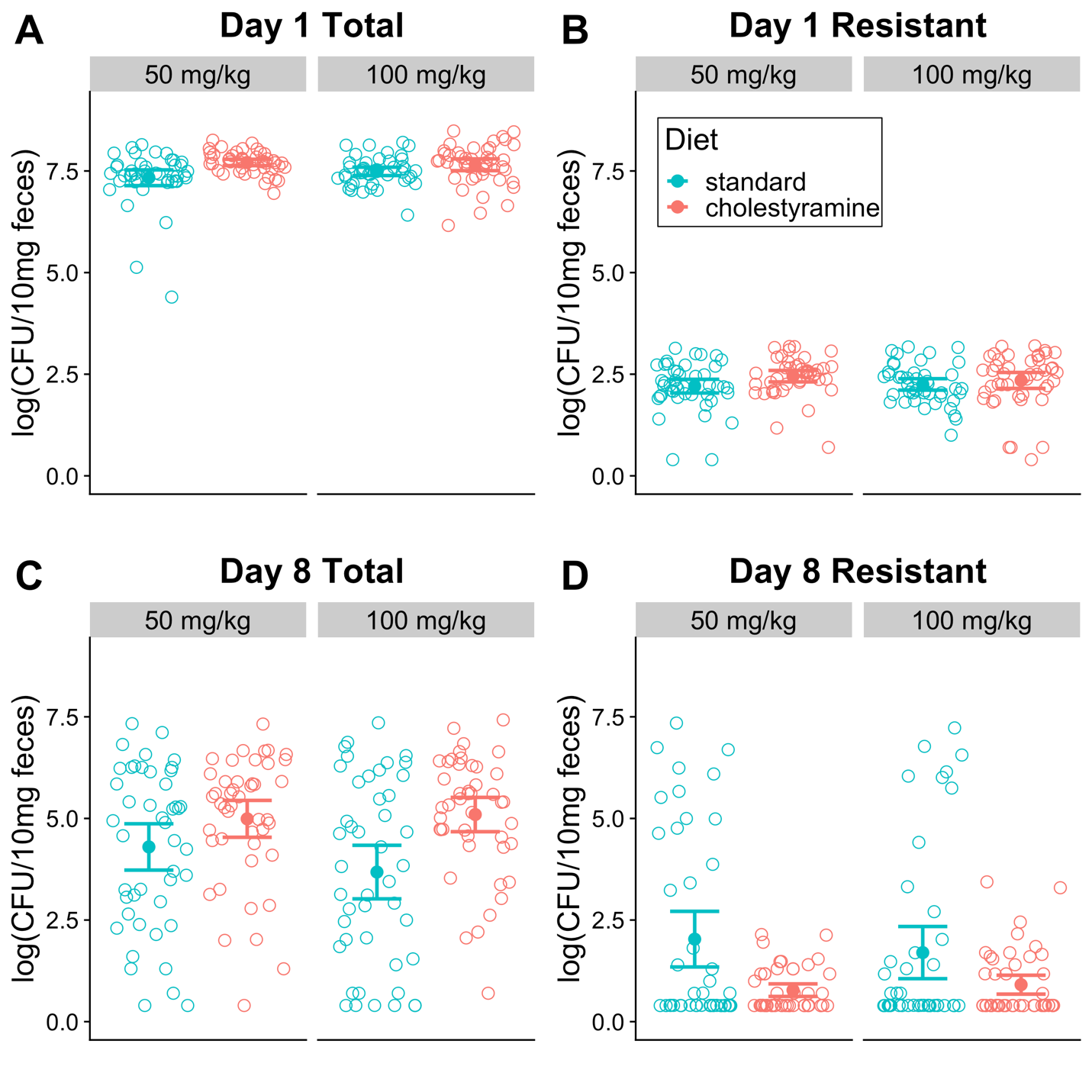


**Supp Fig 1.** **Densities of *E. faecium* in mouse feces during *de novo* emergence experiment.** Each point shows the density of *E. faecium* in a fecal sample from one mouse. Densities were determined by plating on Enterococcosel agar with and without daptomycin supplementation. Mean and 95% CI shown. Samples were analyzed at Day 1 (prior to daptomycin treatment) and Day 8 (post daptomycin treatment) A) Total *E. faecium* densities at Day 1. B) Densities of daptomycin-resistant *E. faecium* at Day 1. C) Total *E. faecium* densities at Day 8. D) Densities of daptomycin-resistant *E. faecium* at Day 8.


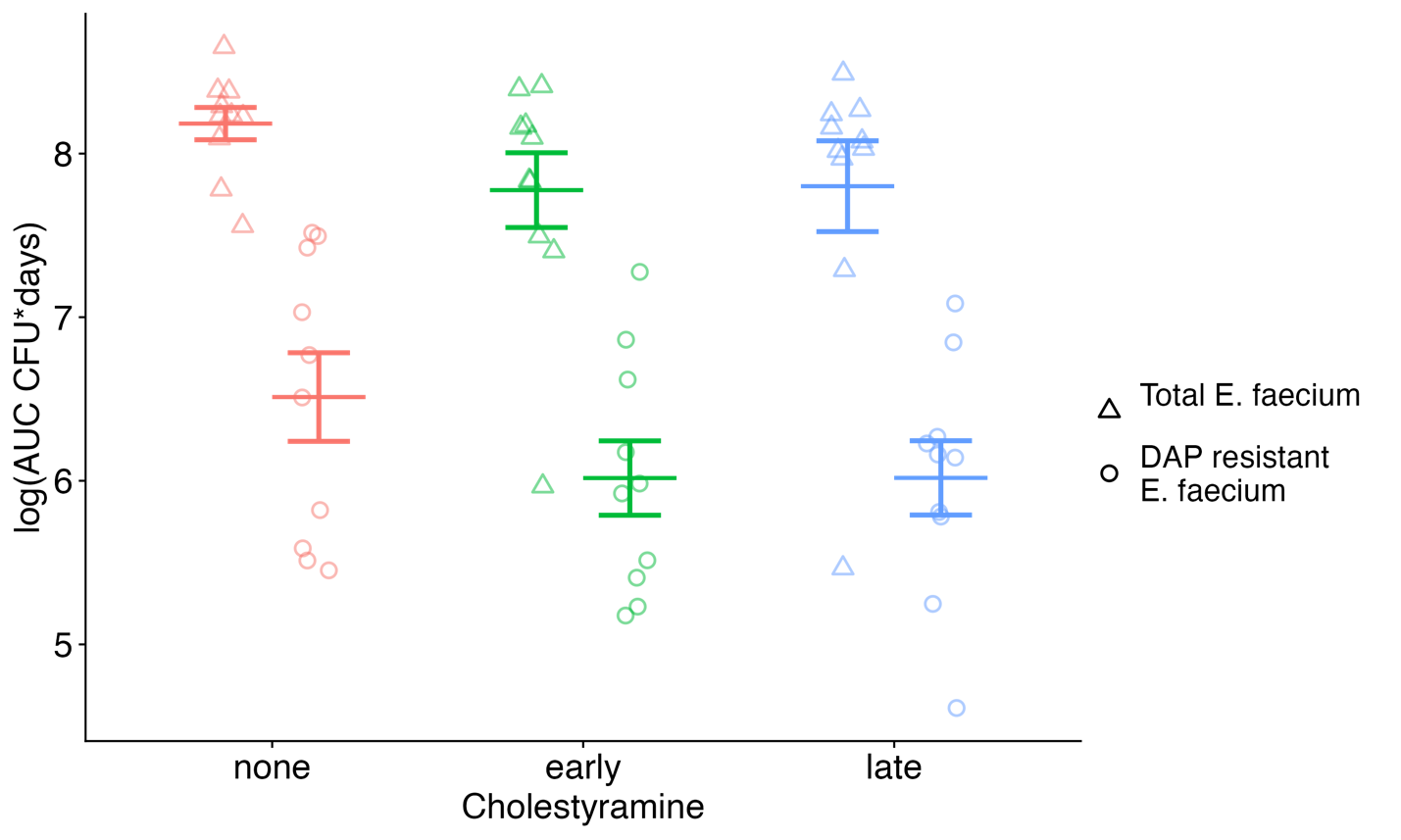


**Supp Fig 2.** **Effect of cholestyramine alone on *E. faecium* densities in experiment 2.** As part of the experiment testing the timing of cholestyramine administration, control mice were provided with cholestyramine in the absence of daptomycin treatment. Mice were provided with a standard diet (no chol), cholestyramine starting at Day 0, or cholestyramine starting at Day 1 (late chol) (N=10 per treatment). Fecal samples were collected at time points throughout the experiment, and densities of *E. faecium* and daptomycin-resistant *E. faecium* were determined by plating. Plot shows the area under the curve (AUC) for the absolute densities of total *E. faecium* and daptomycin-resistant *E. faecium* in fecal samples over the total duration of the experiment. Points indicate AUCs for individual mice for total *E. faecium* (triangles, CFU measured on Enterococcosel plates without daptomycin) and daptomycin-resistant *E. faecium* (circles, CFU measured on Enterococcosel plates with daptomycin). Means and SE are shown (N=10).
